# Pleiotropic Effects of Metformin in Osteoarthritis

**DOI:** 10.3390/life13020437

**Published:** 2023-02-03

**Authors:** Sevdalina Nikolova Lambova

**Affiliations:** 1 Department of Propaedeutics of Internal Diseases “Prof Dr Anton Mitov”, Faculty of Medicine, Medical University of Plovdiv, 4002 Plovdiv, Bulgaria; sevdalina_n@abv.bg; 2 Department in Rheumatology, MHAT “Sveti Mina”, 4002 Plovdiv, Bulgaria

**Keywords:** osteoarthritis, metformin

## Abstract

The involvement of the knee joint is the most common localization of the pathological process in osteoarthritis (OA), which is associated with obesity in over 50% of the patients and is mediated by mechanical, inflammatory, and metabolic mechanisms. Obesity and the associated conditions (hyperglycemia, dyslipidemia, and hypertension) have been found to be risk factors for the development of knee OA, which has led to the emerging concept of the existence of a distinct phenotype, i.e., metabolic knee OA. Combined assessment of markers derived from dysfunctional adipose tissue, markers of bone and cartilage metabolism, as well as high-sensitivity inflammatory markers and imaging, might reveal prognostic signs for metabolic knee OA. Interestingly, it has been suggested that drugs used for the treatment of other components of the metabolic syndrome may also affect the clinical course and retard the progression of metabolic-associated knee OA. In this regard, significant amounts of new data are accumulating about the role of metformin—a drug, commonly used in clinical practice with suggested multiple pleiotropic effects. The aim of the current review is to analyze the current views about the potential pleiotropic effects of metformin in OA. Upon the analysis of the different effects of metformin, major mechanisms that might be involved in OA are the influence of inflammation, oxidative stress, autophagy, adipokine levels, and microbiome modulation. There is an increasing amount of evidence from in vitro studies, animal models, and clinical trials that metformin can slow OA progression by modulating inflammatory and metabolic factors that are summarized in the current up-to-date review. Considering the contemporary concept about the existence of metabolic type knee OA, in which the accompanying obesity and systemic low-grade inflammation are suggested to influence disease course, metformin could be considered as a useful and safe component of the personalized therapeutic approach in knee OA patients with accompanying type II diabetes or obesity.

## 1. Introduction

Osteoarthritis (OA) is the most common joint disease [1] that affects all joint structures with the development of progressive articular cartilage damage, synovial inflammation, and bony changes [2]. OA has complex pathogenesis that may manifest with different joint localization. However, a standardized approach is used in the different forms of OA, and established strategies according to the different pathogenetic mechanisms and phenotypes are lacking. Moreover, currently, there is no disease-modifying drug for OA, and the global results of treatment are unsatisfactory [1]. Combined therapeutic interventions that target specific underlying pathological processes of OA would likely have disease-modifying potential. Targeted and individualized treatment for a specific clinical case is the basis of the modern concept of personalized medicine. A new personalized approach to patients with different OA phenotypes may suggest the opportunity to slow down disease progression.

The pathogenesis of OA is complex with the involvement of different cell types (chondrocytes, synoviocytes, bone cells, inflammatory cells) and up-regulation of multiple mediators such as nitric oxide (NO), prostaglandin E2/PGE2, proinflammatory cytokines (interleukin/IL-1β, TNF-α, IL-6), cartilage-degrading enzymes (matrix metalloproteinases (MMP) 1, 3, 9, and 13 that degrade collagen type II and “aggrecanases”, i.e., a disintegrin and metalloproteinase with thrombospondin motifs (ADAMTS)-4 and -5 that degrade aggrecan) [3,4,5]. Increased expression of insulin-like growth factor/IGF, transforming growth factor/TGF-β in articular cartilage and in osteophytes has been also observed [6].

Pharmacological treatment for OA includes the use of non-steroidal anti-inflammatory drugs, analgesics, and symptomatic slow-acting agents (glucosamine, chondroitin, soy, avocado, and intra-articular hyaluronic acid). Although there is evidence that glucosamine and chondroitin have a chondroprotective effect and can slow the progression of OA, there is currently no drug registered as a disease-modifying agent in OA [7,8,9].

The involvement of the knee joint is the most common localization of the pathological process in OA that is associated with obesity in over 50% of the patients and is mediated by mechanical, inflammatory, and metabolic mechanisms [1]. Obesity and the associated conditions (hyperglycemia, dyslipidemia, and hypertension) have been found to be risk factors for the development of knee OA, which has led to the emerging concept of the existence of a distinct phenotype, i.e., metabolic knee OA [10]. A younger age of onset and absence of OA with other localizations have been noted, as well as an increased level of adipokines in patients with radiologically confirmed knee OA and obesity [11]. In young patients with knee pain, other syndromes should be considered in the differential diagnosis, such as patellar tendinopathy, chondromalacia patellae, patellofemoral OA, pes anserine bursitis, plica syndrome, iliotibial band syndrome, etc. Plain radiography is indicated to rule out knee OA [12,13,14].

Interestingly, the increased risk of obese patients for the development of OA was suggested to be not only a consequence of the biomechanical load on weight-bearing joints but also of hand OA, which suggests an association with the action of systemic factors such as adipokines derived from the dysfunctional adipose tissue [3,15,16]. There is evidence that some adipokines, i.e., leptin and resistin, have catabolic effects on articular cartilage [17,18]. It is increasingly hypothesized that adipocytokines mediate the link between obesity and OA and have a negative effect on joint function and disease progression [10,11].

Combined assessment of markers derived from dysfunctional adipose tissue, markers of bone and cartilage metabolism, as well as high-sensitivity inflammatory markers and imaging, might reveal prognostic signs for metabolic knee OA. Interestingly, it has been suggested that drugs used for the treatment of other components of the metabolic syndrome may also affect the clinical course and retard the progression of metabolic-associated knee OA [1,19,20,21,22,23]. In this regard, significant amounts of new data are accumulating about the role of metformin—a drug, commonly used in clinical practice with suggested multiple pleiotropic effects [1,23].

The aim of the current review is to analyze the current views about the potential pleiotropic effects of metformin in OA.

## 2. Metformin

Metformin is a widely used biguanide with a good safety profile that is the first-line treatment for type II diabetes mellitus, especially in overweight and obese patients after the failure of diet [1,24]. Metformin is an extract of the plant Galega officinalis and has been used in clinical practice for more than 50 years [25]. Metformin has a glucose-lowering effect. In addition, it modulates inflammatory and metabolic factors and leads to weight loss and a decrease in the levels of plasma lipids [1]. The initial dose is 500–850 mg two or three times daily and could be gradually increased to the maximum daily dose of 1000 mg three times daily (3 g).

## 3. Safety Profile of Metformin

The most common adverse effects of metformin are gastrointestinal symptoms, i.e., increased bloating, flatulence, diarrhea, cramps, nausea, and vomiting [24,26], which in some cases may be avoided by a gradual increase in the dose. A serious adverse event is metformin-associated lactic acidosis, which in severe cases may require renal replacement therapy. However, this complication is rare, and its occurrence may be decreased by avoiding metformin administration in patients with a high risk of hypovolemia, sepsis, renal impairment, elderly patients with reduced renal capacity, hypoxic respiratory diseases, and heart failure. In addition, metformin use should be avoided preoperatively for 24–78 h as well as before intravenous injection of contrast media, as it may increase the risk of nephropathy. Another consideration for clinical practice is the development of vitamin B12 deficiency during metformin treatment and reduced folate levels [24,26]. The key advantage of metformin is that it does not cause hypoglycemia [27], including in normoglycemic patients [28].

## 4. Mechanism of Action of Metformin

Metformin is a hypoglycemic agent that inhibits liver gluconeogenesis, increases insulin sensitivity, promotes the uptake of glucose in peripheral tissues, i.e., skeletal muscles and fat tissue, and delays the intestinal absorption of glucose. It influences the incretin axis via an increase in the level of the glucagon-like peptide-1 that elevates insulin secretion and reduces the secretion of glucagon in response to glucose [25,29,30]. Metformin acts in an AMPK (adenosine monophosphate/AMP-activated protein kinase)-dependent and AMPK-independent pathway [30]. Metformin penetrates the mitochondrial inner membrane and directly inhibits the mitochondrial respiratory chain complex 1 [30,31]. It suppresses mitochondrial respiration, lysosomal-related pathways, and the expression of gluconeogenesis-related enzymes. It increases the level of AMP (AMP/adenosine triphosphate/ATP ratio) that activates AMPK [30]. AMPK modulates metabolism according to the energy demand and responds to changes in AMP [32].

It has been hypothesized that AMPK activity may support cartilage homeostasis, and decreased expression of AMPK was found in OA articular chondrocytes [33]. In this regard, AMPK could be a target for OA treatment. Mitochondrial injury is suggested to be associated with the pathological process in OA [31].

In mouse chondrocytes stimulated by IL-1β to induce an OA model, decreased expression of sirtuin 3 was found. Sirtuin 3 protects mitochondria against oxidative stress, and its downregulation is associated with mitochondrial damage and oxidative stress due to the increased production of reactive oxygen species. In an in vitro model, Wang et al. (2019) have demonstrated that treatment with metformin increased sirtuin 3, decreased the production of reactive oxygen species in the mitochondria, and reduced the loss of cell viability [34].

In a cell culture of IL-1β-induced ATDC5 cells, it has been demonstrated that metformin blocks the NF-κB pathway via activation of AMPK [35]. In OA, the chondrocytes shift to a degradative phenotype, in which NF-κB triggers the secretion of MMPs (1,2,3,7,8,9,13) and aggrecanases. Osteoarthritic chondrocytes also express a variety of NF-κB-mediated proinflammatory cytokines such as TNF-α, IL-1β, IL-6, receptor activator of NF-κB (RANK ligand/RANKL). NF-κB mediates articular damage through the induction of nitric oxide, cyclooxygenase 2, and PGE2, which induce the synthesis of catabolic factors, cartilage inflammation, and chondrocyte apoptosis [36].

## 5. Suggested Pleiotropic Effects of Metformin and Other Clinical Uses

Apart from its hypoglycemic effects in diabetes type 2, various pleiotropic effects of metformin have been suggested, i.e., cardiovascular protection, beneficial effects on obesity, musculoskeletal and reproductive diseases, cancer, and aging [27,30] (Table 1).

Metformin has proven efficacy in reducing body mass index (BMI) in overweight and obese adolescents and adults without diabetes. Metformin treatment should be accompanied by appropriate diet and lifestyle changes. In a recent systematic review and meta-analysis by Hui F et al. (2019), it was concluded that the optimal dose in obesity is 1000 mg/day of metformin for 3 months in adolescents and 3000 mg daily for 6 months in adults [27]. As it does not induce hypoglycemia, it could be safely administered to normoglycemic subjects.

Improved cardiovascular outcomes have been documented in patients with diabetes mellitus type II [39] and are suggested to be related to improved glycemia, beneficial effects on endothelial function, hemostasis, i.e., inhibition of platelet aggregation and blood viscosity, reduced levels of coagulation factors VII, XIII, von Willebrand factor, and enhanced fibrinolysis [39,40,41].

Another clinical setting in which metformin has shown clinical benefit is polycystic ovarian syndrome, which is characterized by insulin resistance and hyperinsulinemia. Metformin decreases insulin levels and, as a consequence, lowers circulating total and free androgen levels, improving ovulation and pregnancy rates [28]. Metformin was also found to be effective and safe for improving polycystic ovarian syndrome-associated acne [38].

A potential role of metformin in osteopenia and osteoporosis has been suggested due to beneficial effects on bony tissue [37] based on the observation of its direct action on osteoblasts through activation of AMPK resulting in osteoblasts differentiation, proliferation, and bone matrix synthesis [42], inhibition of RANKL signaling and increase in osteoprotegerin expression by osteoblasts that leads to reduced osteoclast number and prevention of bone loss [43]. Moreover, lower fracture risk was observed in diabetic patients treated with metformin as compared with non-metformin users and those treated with other antidiabetic agents [44].

A recent analysis by Yu H. et al. of 21 systematic reviews and meta-analyses that evaluated the effect of metformin on cancer incidence and survival has led to the conclusion that there is strong evidence for the association between metformin use and decreased pancreatic cancer incidence and highly suggestive evidence for the association between metformin use and improved colorectal cancer overall survival. Only suggestive evidence was found for all cancer incidence and overall survival, including the overall survival of breast, lung, and pancreatic cancer and the incidence of colorectal cancer and liver cancer [29]. An increase in the expression of the tumor-suppression gene p53 was observed through the activation of AMPK in hepatoma cell culture treated with metformin [45]. Other mechanisms have also been suggested, such as a decrease in IGF-1, which is associated with the stimulation of cell mitogenesis [29,46].

## 6. Pleiotropic Effects of Metformin in Osteoarthritis

**Figure 1:**
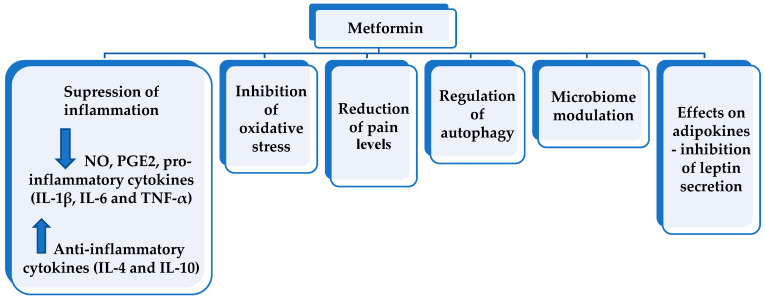
Pleiotropic effects of metformin in OA [26,30,47,48,49,50,51,52,53].

In a recent review by Song et al. (2022), major effects of metformin in OA were suggested, i.e., the influence of inflammation, oxidative stress, autophagy, and pain levels that are related to the activation of AMPK, which is a major regulator of cell metabolism and is involved in various signaling pathways [30]. Additional mechanisms of metformin that might exert pleiotropic effects in OA include microbiome modulation and the influence of adipokine levels (Figure 1).

### 6.1. Anti-Inflammatory Effect

In in vitro experiments, it has been demonstrated that metformin reduced the production of NO, PGE2, and pro-inflammatory cytokines (IL-1β, IL-6, and TNF-α) by inhibition of NF-κB translocation in macrophages and this effect was dose dependent. In addition, metformin increased the protein expression of anti-inflammatory cytokines, i.e., IL-4 and IL-10 [47]. The anti-inflammatory effect of metformin has also been confirmed in humans. In patients with diabetes mellitus type II, a significant reduction in serum levels of IL-6 and TNF-α was found [48].

### 6.2. Effects on Oxidative Stress

Oxidative stress has been involved in the pathogenesis of both diabetes type II and OA. Inflammatory mediators, such as IL-1β, IL-6, and TNF-α, induce the production of reactive oxygen species in OA and stimulate the expression of matrix-degrading enzymes [54]. A reduction in oxidative stress in metformin users with type II diabetes was registered via measurement of oxidative stress markers such as advanced oxidation protein products [49].

### 6.3. Effects on Pain Level

AMPK activation is suggested to be involved in analgesia [55,56]. It has been demonstrated that the AMPK signaling pathway is related to mammalian target of rapamycin complex 1 (mTORC1), which regulates different cellular functions including translation in response to nutrients (mainly amino acids), cell proliferation, and growth [32,57]. There is evidence that mTORC1 signaling participates in the transmission of both neuropathic and inflammatory pain [57]. AMPK activation inhibits mTORC1 signaling [32]. It has also been found that activation of AMPK attenuates inflammatory pain by suppressing NF-kB in activated macrophages and subsequent inhibition of the overexpression of IL-1β. It has been suggested that blocking IL-1 receptor of IL-1β is involved in inflammatory pain relief [56]. In a mouse animal model, it has been demonstrated that systemic administration of metformin with activation of AMPK inhibited incision-evoked mechanical hypersensitivity and hyperalgesic priming induced by injection of PGE2 [58]. Hyperalgesic priming is characterized by increased nociceptor sensitization induced by pro-nociceptive mediators (such as PGE2) and represents a model of transition from acute to chronic pain [59]. Thus, it has been concluded that metformin could be effective in reducing the transition to a chronic pain state [55].

### 6.4. Regulation of Autophagy

Autophagy is a catabolic pathway conserved in eukaryotes for rapid elimination of large unwanted structures such as aberrant protein aggregates, damaged organelles, and invading pathogens. The materials that should be degraded undergo sequestration in the lysosomes by double-membrane vesicles called autophagosomes [60]. A decrease in the autophagic clearance capacity of the cells could be observed during aging, which may favor chondrocyte apoptosis. AMPK and mTOR are important regulators of autophagy initiation. In this regard, metformin may also exert chondroprotective effects by upregulation of autophagy [30]. An increased expression of autophagy marker LC3 was observed under treatment with metformin [50]. Being an AMPK activator, it has been hypothesized that metformin may suppress chondrocyte apoptosis [30]. Mitophagy is a specific autophagic elimination of damaged mitochondria that is regulated by the kinase phosphatase and tensin homolog—induced putative kinase protein 1 (PINK1)/Parkin pathway. It has been demonstrated in an in vitro study that metformin promotes PINK1/Parkin-mediated mitophagy in chondrocytes. Metformin reduced IL-1β-induced expression of MMP-3 and MMP-13 and enhanced the anabolic marker collagen Ⅱ. These effects are suggested to be mediated by the upregulation of the PINK1/Parkin pathway [34].

### 6.5. Microbiome Modulation

The gut microbiome is suggested to contribute to different metabolic disorders, i.e., obesity, diabetes, metabolic syndrome, autoimmune disorders, inflammatory bowel disease, liver disease, cancer, and possibly the aging process. Different factors that lead to disturbances of the microbiome (dysbiosis), such as obesity, a high-fat diet, and antibiotic use, are associated with the release of the proinflammatory microbial lipopolysaccharide in the circulation [51]. Aging is suggested to be associated with a subclinical chronic inflammatory process, i.e., inflamm-aging [26,61]. Moreover, an increase in intestinal permeability with aging could be observed [26]. Aging and obesity are the two strongest nongenetic risk factors for OA. Both conditions are characterized with changes in the gut microbiome that might be related to the development of OA. Moreover, microbiome disbalance might be a potential trigger of chronic innate immune activation in OA [62,63].

Until recently, cartilage was thought to be a sterile tissue. A novel study by Dunn C et al. (2020) has revealed the presence of a microbial DNA signature in patients with knee and hip OA and in mice predisposed to the development of OA. Using 16S ribosomal RNA gene deep sequencing, the authors identified cartilage microbial DNA signatures in mouse knee tissues, which in OA-resistant mice were similar to disease-free human control tissue, whereas in OA-susceptible mice were similar to human OA tissue. The authors also analyzed sections of hip and knee cartilage obtained from patients undergoing joint replacement for end-stage primary OA and control cartilage tissue from cadavers without clinical and histological data for OA. In OA specimens, members of the phylum Proteobacteria were enriched, specifically the class Betaproteobacteria, while among the controls, the phylum Bacteroidetes and the class Alphaproteobacteria were enriched. A significant increase in microbial DNA from Gram-negative organisms in human cartilage samples from OA patients was found as compared to disease-free controls. Interestingly, different DNA signatures were detected in human cartilage from knee and hip joints both in OA and in controls [63].

Obesity-associated inflammation is triggered by lipopolysaccharides derived from the gut microbiota. In an animal model of OA in rats, associations between gut microbiota, systemic lipopolysaccharide levels, serum and local inflammatory changes, and joint damage were assessed in a high-fat diet-induced obese rat model as compared with a standard control diet group. A distinct inflammatory signature in synovial fluid and serum in rats with diet-induced obesity was found that was more pronounced in the synovial fluid. The quantity of the gut microbes *Lactobacillus* spp. was negatively and *Methanobrevibacter* spp. positively associated with histological changes and proinflammatory mediators in serum and synovial fluid [64].

It has been suggested that metformin modulates bacterial growth and has a positive effect on microbiome composition, maintaining a healthy phenotype [26,51]. In in vitro experiments, it has been demonstrated that metformin alters microbial folate and methionine metabolism in some *E. coli* strains but its action does not depend on *E. coli* lipopolysaccharide subtype. Metformin-induced alteration of microbial metabolism might contribute to its therapeutic efficacy and also to its side effects, i.e., folate deficiency and gastrointestinal upset [26].

### 6.6. Decrease in Leptin Level

The existence of receptors for leptin in chondrocytes and an increase in its level in serum and synovial fluid in OA suggest that leptin may be involved in the pathogenesis of OA, connecting obesity to OA [65,66]. In animal studies, it has been demonstrated that leptin has a catabolic effect on articular cartilage and leads to a significant increase in both gene and protein levels of MMP-2 and 9, cathepsin D, and a decrease in basic fibroblast growth factor [18]. In an in vitro study, it has been demonstrated that metformin has a direct, selective interaction with adipocytes, in which it inhibits leptin secretion via a mitogen-activated protein kinase signaling pathway [52]. A significant decrease in leptin level was also detected in vivo in adolescents after 8-week treatment with metformin [53].

### 6.7. Disease-Modifying Potential of Metformin in Osteoarthritis—Evidence from Animal Models, In Vitro Experiments, and Clinical Trials in Humans

There is evidence from animal models, in vitro experiments, and clinical trials in humans that metformin can slow the progression of OA by modulating inflammatory and metabolic factors (Table 2). Thus, further evaluation of its potential as a disease-modifying drug and a component of combination personalized therapy, especially for metabolic type knee OA, deserves attention.

In an experimental rat model of OA induced with sodium iodoacetate, a positive therapeutic effect was registered when mesenchymal stem cells derived from adipose tissue and treated with metformin were administered. Rats with experimental OA were divided into three groups: a control group and two groups treated with adipose mesenchymal stem cells with or without metformin stimulation, respectively. An increased migration potential of mesenchymal cells treated with metformin was observed, affecting their immunoregulatory properties and suppressing the pro-inflammatory and catabolic mediators involved in the pathogenesis of OA. Decreased expression of IL-1β and IL-6 was found in mesenchymal stem cells from adipose tissue stimulated with metformin. In IL-1β-stimulated OA chondrocytes, metformin-stimulated mesenchymal stem cells inhibited the mRNA level for collagen type X, vascular endothelial growth factor, and MMP-1, 3 and 13 and increased the expression of tissue inhibitors of MMP-1 and 3. Limb nociception was evaluated by measuring the paw withdrawal latency and threshold. The antinociceptive and chondroprotective effects of mesenchymal stem cells treated with metformin were more pronounced compared to those of unstimulated cells [67]. In our own study in a mouse model of OA, decreased cartilage degeneration was demonstrated after treatment with metformin, and the effect was more pronounced in the group treated with the combination of metformin and the biphosphonate—alendronate. In this regard, combination therapy that also includes bisphosphonates, which are inhibitors of bone resorption in OA phenotypes with increased bone remodeling, could also be beneficial [23]. Activation of autophagy and downregulation of chondrocyte apoptosis were observed after intraarticular administration of metformin in a mouse model of OA [68].

It has been found that metformin protects chondrocytes against IL-1β-induced injury [35]. A decreased expression of AMPK in OA articular chondrocytes could be found, which suggests that maintenance of AMPK activity may support cartilage homeostasis [33]. Thus, AMPK has been considered a target for OA suppression [31]. In a cell culture of human chondrocytes from patients undergoing knee arthroplasty for end-stage OA, metformin reduced the expression of the catabolic genes ADAMTS-5 and MMP-1 [70]. In human IL-1β-stimulated OA chondrocytes, metformin reduced catabolic factor gene expression (MMP-1, 3, 13), increased mRNA levels of tissue inhibitors of MMP-1 and 3, and promoted autophagy [69].

In a clinical trial in patients with symptomatic, radiologically confirmed knee OA, a greater reduction in serum levels of IL-1β, IL-8, and TNF-α was found in patients treated with metformin and meloxicam compared to those receiving meloxicam alone over a 12-week period [71]. In patients with OA and type II diabetes mellitus, the combined administration of a COX-2 inhibitor and metformin resulted in a reduced incidence of joint replacement compared with patients receiving a COX-2 inhibitor alone during a 10-year period of follow-up [72].

Wang et al. (2019) reported data from Osteoarthritis Initiative participants with delayed OA progression at 4-year follow-up in patients with radiographically confirmed OA of the knee joint (radiographic stage ≥ 2 according to the Kellgren–Lawrence scale) who had concomitant obesity with a BMI ≥ 30 kg/m^2^ and were taking metformin. Fifty-six patients were on metformin therapy at baseline and at the first and second years of the follow-up, and 762 patients were non-users of metformin. Assessment of articular cartilage volume of the femur and tibia was performed via magnetic resonance imaging at baseline and at the fourth year. The rate of medial cartilage volume loss was found to be lower in patients taking metformin compared to metformin non-users (0.71% vs. 1.57% per year), with a difference of 0.86% per year, which is statistically significant (*p* = 0.02) after adjustment for other factors such as age, gender, BMI, pain score, radiological stage according to the Kellgren–Lawrence scale, self-reported diabetes, and change in body weight over a period of 4 years. These data suggest a possible long-term positive effect of metformin in patients with OA and obesity, and a possible disease-modifying potential of the drug [1].

There is an increasing amount of evidence from in vitro studies, animal models, and clinical trials that metformin can slow OA progression due to its anti-inflammatory effects (decrease in the level of proinflammatory cytokines and leptin), anti-oxidative effects, upregulation of autophagy, and modulation of the microbiome. Considering the contemporary concept about the existence of metabolic type knee OA, in which accompanying obesity and systemic low-grade inflammation are suggested to influence disease course, metformin could be considered a useful and safe component of the personalized therapeutic approach in knee OA patients with accompanying type II diabetes and/or obesity that is suggested to inherit disease-modifying potential in knee OA together with its positive effects on body weight in obese patients.

## Figures and Tables

**Table 1:** Suggested benefits from metformin use due to its pleiotropic effects.

Indication for Metformin Treatment	Benefits from Metformin Use Due to Its Pleiotropic Effects	References
Diabetes mellitus type II	Obesity Cardiovascular protection Polycystic ovarian syndrome d Musculoskeletal diseases—osteopenia, osteoporosis, osteoarthritis Cancer Aging	[27,29,37,38]

**Table 2:** Major studies that present results from animal models, in vitro experiments, and clinical trials in humans about effects of metformin in OA.

Study (Author’s Group, Year)	Study Design	Conclusions about Pleiotropic Effects of Metformin in OA
*I*. Animal models		
Park M-J et al. (2019) [67]	Rats with experimental OA were treated with adipose mesenchymal stem cells with or without metformin stimulation.	The antinociceptive and chondroprotective effect of mesenchymal stem cells treated with metformin was superior compared to that of unstimulated cells.
Belenska-Todorova L et al. (2021) [23]	Mouse model of OA; metformin administered orally.	Decreased cartilage degeneration was confirmed histologically after treatment with metformin compared with controls.
Wang C et al. (2020) [68]	Mouse model of OA; metformin administered intraarticularly	Intraarticular administration of metformin led to improved histological findings, i.e., decreased cartilage damage, increased proteoglycan expression, and reduced the synovium thickness.
Na HS et al. (2021) [69]	OA in rats; metformin was administered orally.	Histological evidence of a decrease in cartilage destruction after treatment with metformin
*II*. In vitro experiments		
Zhang M et al. (2020) [35]	Cell viability of ATDC5 cells was assessed after treatment with metformin. Effects of metformin on osteoarthritic changes induced in ATDC5 cells with IL-1β application.	Metformin promoted proliferation of ATDC5 cells; metformin reversed partly IL-1β-induced changes, i.e., the increase in the level of proteins of proinflammatory cytokines IL-6 and TNF-α.
Schadler P et al. (2021) [70]	Primary chondrocytes from 14 adult female patients undergoing knee arthroplasty for end-stage OA were cultivated and stimulated with metformin. Matrix gene expression was analyzed via polymerase chain reaction.	Metformin decreased the expression of catabolic genes ADAMTS5 and MMP-1.
Na HS et al. (2021) [69]	Human IL-1β-stimulated OA chondrocytes treated with metformin.	Metformin reduced catabolic factor gene expression (MMP-1, 3, 13), increased mRNA levels of tissue inhibitors of MMP-1 and 3 and increased expression of autophagolysosome markers.
*III*. Human studies		
Mohammed MM et al. (2014) [71]	68 patients with symptomatic, radiologically confirmed knee OA; 12-week follow-up.	A greater reduction in serum levels of IL-1β, IL-8, and TNF-α was found in patients treated with metformin (2 × 500 mg daily) and meloxicam compared to those receiving meloxicam.
Lu CH et al. (2018) [72]	Patients with OA and diabetes mellitus type 2; 968 on metformin, 1936 without metformin; 10-year period of follow-up; the rate joint replacement surgery was assessed.	Significantly lower rate of joint replacement surgery was registered in metformin users at the end of follow-up compared with the control group (*p* = 0.003).
Wang Y et al. (2019) [1]	Osteoarthritis Initiative data; 56 patients on metformin; 762 patients were non-users of metformin.	Assessment of articular cartilage volume of the femur and tibia performed via MRI at baseline and at the fourth year has shown that the rate of medial cartilage volume loss was lower in patients taking metformin compared to metformin non-users (*p* = 0.02).

## Data Availability

Not applicable.
